# Computational Prediction of acyl-coA Binding Proteins Structure in *Brassica napus*

**DOI:** 10.1371/journal.pone.0129650

**Published:** 2015-06-11

**Authors:** Nadia Haingotiana Raboanatahiry, Guangyuan Lu, Maoteng Li

**Affiliations:** 1 College of Life Science and Technology, Huazhong University of Science and Technology, Wuhan, 430074, China; 2 Oil Crops Research Institute, Chinese Academy of Agricultural Sciences, Wuhan, Hubei, 430062, China; 3 Hubei Collaborative Innovation Center for the Characteristic Resources Exploitation of Dabie Mountains, Huanggang, 435599, China; National Key Laboratory of Crop Genetic Improvement, CHINA

## Abstract

Acyl-coA binding proteins could transport acyl-coA esters from plastid to endoplasmic reticulum, prior to fatty acid biosynthesis, leading to the formation of triacylglycerol. The structure and the subcellular localization of acyl-coA binding proteins (ACBP) in *Brassica napus* were computationally predicted in this study. Earlier, the structure analysis of ACBPs was limited to the small ACBPs, the current study focused on all four classes of ACBPs. Physicochemical parameters including the size and the length, the intron-exon structure, the isoelectric point, the hydrophobicity, and the amino acid composition were studied. Furthermore, identification of conserved residues and conserved domains were carried out. Secondary structure and tertiary structure of ACBPs were also studied. Finally, subcellular localization of ACBPs was predicted. The findings indicated that the physicochemical parameters and subcellular localizations of ACBPs in *Brassica napus* were identical to *Arabidopsis thaliana*. Conserved domain analysis indicated that ACBPs contain two or three kelch domains that belong to different families. Identical residues in acyl-coA binding domains corresponded to eight amino acid residues in all ACBPs of *B*. *napus*. However, conserved residues of common ACBPs in all species of animal, plant, bacteria and fungi were only inclusive in small ACBPs. Alpha-helixes were displayed and conserved in all the acyl-coA binding domains, representing almost the half of the protein structure. The findings confirm high similarities in ACBPs between *A*. *thaliana* and *B*. *napus*, they might share the same functions but loss or gain might be possible.

## Introduction

The function of proteins depends greatly on their structure. A small change or difference in their structure may alter the original function and may cause harmful effects. The acyl-coA binding proteins (ACBPs) are highly conserved across species and paralogues have evolved leading to multiple important functions [1]. In fact, ACBPs could transport fatty acid to endoplasmic reticulum after their biosynthesis into the plastid [2, 3, 4]. These fatty acids are involved in the biosynthesis of triacylglycerol (TAG), the most important compound of food or biodiesel oils. In *Brassica napus*, small ACBPs could effectively alter TAG composition in seeds [5]. *B*. *napus* is an important crop which is closely related to *A*. *thaliana*, they both belong to *Brassicaceae* family. *A*. *thaliana* ACBPs (*At*ACBPs) are divided in four classes according to their structure which could define their functions, expressions, subcellular localizations and acyl-coA binding affinities: Class I represents the small *At*ACBPs which have 92 amino acids and weigh 10.4 KDa. Class II *At*ACBPs contain ankyrin repeats domain, they are widely called ACBP1 and ACBP2. ACBP1 contain 338 amino acids (37.5 KDa) and ACBP2 have 354 amino acids (38.5 KDa). Class III are the large *At*ACBPs which have 362 amino acids (39.3 KDa) and Class IV are the kelch motif containing ACBP4 and ACBP5. ACBP4 have 668 amino acids (73.2 KDa) and ACBP5 have 648 amino acids (71 KDa) [6, 7, 8, 9, 10, 11, 12]. The structure of ACBP was investigated in bovine: four alpha-helixes jointed by hydrophobic interactions and exposed an up-down-down-up direction were demonstrated [13]. The ligand-binding site in *A*. *thaliana* was highlighted showing conserved residues of common ACBPs across the species [6]. This ligand-binding site is divided into three subsites: one for the acyl part of the ligand, one for the adenine ring and one for the 3’-phosphate [14]. The adenine rings and the acyl part of the ligand are attached to the ACBP through non-polar connections [15] and the 3'-phosphate interacts with the ACBP with salt bridges and hydrogen bond [16]. Therefore, a single binding site is dedicated to acyl-coA esters only; the bond is strong with high affinity [10, 17, 18]. This binding affinity highly depends on the length of the acyl chain with a preference for more than eight carbon atoms in acyl-CoA esters [19], but the most preferable are those that have twelve to twenty carbon atoms [20]. In *A*. *thaliana*, diverse ligand specificities in each class of ACBP and their different subcellular localizations make them distinct from one to another and enhance them to specialized functions. Studies related to the structure of ACBPs in *B*. *napus* (*Bn*ACBPs) or their subcellular localization were limited and were focused especially on the small 10 KDa *Bn*ACBP. The current work aims to predict the structure of all four classes of *Bn*ACBPs using bioinformatics approaches. The gene structure, the physicochemical parameters, the conserved domains and conserved residues were analyzed. Predictions on secondary and tertiary structure of each *Bn*ACBP, with their subcellular localization were carried out.

## Methods

### Research of *Bn*ACBPs

Small, large and ankyrin repeats *Bn*ACBPs were acquired from Genoscope Database (http://www.genoscope.cns.fr/brassicanapus/) [21]. They were identified as BnaAnng25690D, BnaA05g36060D, BnaA08g07670D and BnaCnng15340D in small *Bn*ACBPs, BnaA02g10270D, BnaA01g16660D, BnaC02g44810D and BnaC01g20440D in ankyrin repeats *Bn*ACBPs, BnaA01g13710D, BnaA03g46540D, BnaC01g16110D and BnaC07g38820D in large *Bn*ACBPs. Kelch motif *Bn*ACBPs were cloned, related sequences are available on the NCBI database (GenBank: Ais76194 to Ais76201). Reports pertaining to the identification and characterizations of *Bn*ACBPs were submitted to BMC Genomics journal.

### Physicochemical parameters analysis

The protein size and the intron-exon description were obtained from the same Genoscope Database. Physicochemical parameters including molecular weight, hydropathicity, isoelectric point (pI) and amino acid composition were analyzed using the ProtParam tool from ExPASy (http://www.expasy.org/) [22].

### Identification of conserved domains and conserved residues

Identification of the conserved domains was performed using SMART (http://smart.embl-heidelberg.de/) [23] and annotated using Pfam (http://pfam.xfam.org/) [24]. The analysis of conserved residues was made using Vector NTI multiple alignments.

### Secondary structure analysis

Secondary structure analysis was carried out using two different tools: PBIL GOR4 (https://npsa-prabi.ibcp.fr/cgi-bin/npsa_automat.pl?page=npsa_gor4.html) [25] and PSIPRED (http://bioinf.cs.ucl.ac.uk/PSIPRED/) [26, 27].

### Tertiary structure analysis

Tertiary structures of ACBPs were predicted using Phyre2 (http://www.sbg.bio.ic.ac.uk/phyre2/html) [28], and they were analyzed using VAST from NCBI database (http://www.ncbi.nlm.nih.gov/Structure/VAST) [29].

### Structure validation

Ramachandran plots [30, 31] were generated using UCSF Chimera software (http://www.cgl.ucsf.edu/chimera) [32].

### Subcellular localization prediction

Predicted subcellular localizations of ACBPs were made using TargetP 1.1 (http://www.cbs.dtu.dk/services/TargetP-1.1) [33] and MultiLoc2 (http://abi.inf.uni-tuebingen.de/Services/MultiLoc2) [34].

## Results

### Physicochemical parameters of *Bn*ACBPs

Physicochemical parameters of *Bn*ACBPs are summarized in Table 1. Analysis showed that the small *Bn*ACBPs weighed 10.03 KDa (BnaAnng25690D) to 10.185 KDa (BnaA08g07670D). Ankyrin repeats *Bn*ACBPs had a weight of 37.204 KDa (BnaC02g44810D) to 40.119 KDa (BnaA01g16660D). Large *Bn*ACBPs weighed 39.285 KDa (BnaC07g38820D) to 41.272 KDa (BnaA01g13710D) and kelch motif *Bn*ACBPs weighed 72.696 KDa (Ais76201) to 73.197 KDa (Ais76200). Additionally, the intron-exon structures of *Bn*ACBP genes were analyzed (Table 1). Analysis indicated that small *Bn*ACBPs contained 3 introns and 4 exons. 5 introns and 6 exons (BnaA02g10270D, BNnaA01g16660D, BnaC02g44810D) or 6 introns and 7 exons (BnaC01g20440D) were detected in ankyrin repeats *Bn*ACBPs. 2 introns and 3 exons (BnaA03g46540D, BnaC01g16110D, BnaC07g38820D) or 3 introns and 4 exons (BnaA01g13710D) were identified in large *Bn*ACBPs. Otherwise, kelch motif *Bn*ACBPs contained 17 introns and 18 exons.

**Table 1:** Physicochemical parameters of *Bn*ACBPs.

TYPE		DESCRIPTION			PHYSICOCHEMICAL PARAMETERS		
		Size (Aa)	Introns	Exons	Weight (kDa)	pI	Hydropathicity
Small	BnaAnng25690D	90	3	4	10.03	5.4	-0.493
	BnaA05g36060D	92	3	4	10.171	5.4	-0.528
	BnaA08g07670D	92	3	4	10.185	5.13	-0.522
	BnaCnng15340D	92	3	4	10.176	5.18	-0.532
	BnaA02g10270D	342	5	6	37.626	4.55	-0.449
Ankyrin	BnaA01g16660D	364	5	6	40.119	4.52	-0.462
repeats	BnaC02g44810D	339	5	6	37.204	4.61	-0.471
	BnaC01g20440D	364	6	7	40.08	4.54	-0.485
Large	BnaA01g13710D	381	3	4	41.272	4.12	-0.287
	BnaA03g46540D	362	2	3	39.435	4.19	-0.332
	BnaC01g16110D	364	2	3	39.894	4.14	-0.326
	BnaC07g38820D	361	2	3	39.285	4.17	-0.327
	Ais76194	666	17	18	73.013	5.24	-0.515
	Ais76195	667	17	18	73.087	5.17	-0.521
	Ais76196	665	17	18	72.906	5.16	-0.535
Kelch	Ais76197	667	17	18	72.716	5.67	-0.436
motif	Ais76198	667	17	18	72.709	5.67	-0.398
	Ais76199	666	17	18	73.079	5.1	-0.518
	Ais76200	667	17	18	73.197	5.17	-0.53
	Ais76201	665	17	18	72.696	5.13	-0.538

The hydropathicity and isoelectric point of *Bn*ACBPs were estimated (Table 1). All ACBP genes had hydropathicity value below 0 with an average value of -0.518, -0.466, -0.318 and -0.498 for small *Bn*ACBPs, ankyrin repeats *Bn*ACBPs, large *Bn*ACBPs and kelch motif *Bn*ACBPs, respectively. In Addition, the low value of isoelectric points were estimated in all four classes of *Bn*ACBP, with an average value of 5.27, 4.55, 4.15 and 5.28 in small *Bn*ACBPs, ankyrin repeats *Bn*ACBPs, large *Bn*ACBPs and kelch motif *Bn*ACBPs, respectively. Amino acid composition analysis revealed that these *Bn*ACBPs contained more negative residues than positive residues (S1 Table). For instance, the ratio of negative residues (Asp + Glu) and positive residues (Lys + Arg) were respectively of 23.4% and 7.8% in large *Bn*ACBP (BnaA01g13710D). These *Bn*ACBPs' high frequency of amino acid residues was Ala (~12%) and Lys (~12%) in small *Bn*ACBPs, Ala (~12%) and Glu (~9%) in ankyrin repeats *Bn*ACBPs, Glu (~17%) in large *Bn*ACBPs and Ser (~11%) in kelch motif *Bn*ACBPs. These results indicated the hydrophilic characteristic of *Bn*ACBPs besides their acidic pI.

### Conserved domains of *Bn*ACBPs

**Fig 1:**
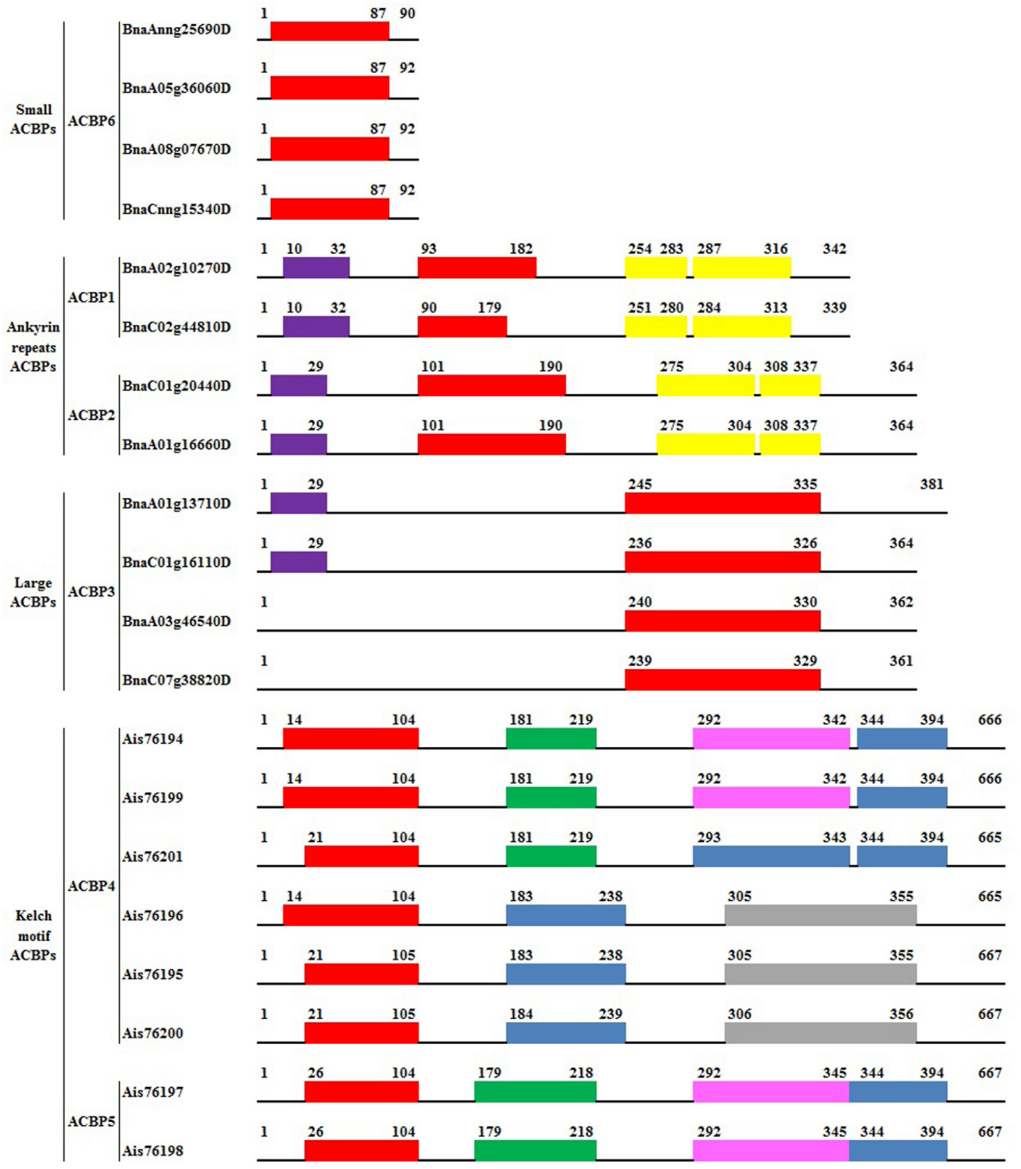
Domain architecture of *Bn*ACBPs. Domains were analyzed using SMART and annotated from Pfam. The numbers indicate residues position. The length of each ACBP are indicated at the end of each structure. The ACBD are labeled in red (PF00887). The ankyrin repeats are labeled in yellow (PF00023). Transmembrane domains are labeled in purple. Kelch motif domains are in green (PF13854), pink (PF13964), blue (PF13418) and grey (not found on Pfam).

Conserved domains of *Bn*ACBPs were analyzed with SMART, focusing on their location and their taxonomy. Domain architecture is shown in Fig 1. The location of domains in each *Bn*ACBP differed from one to another even within the same class. In all ACBPs, the acyl-coA binding domain (ACBD) belonged to PF00887, but their location differed greatly. In small ACBPs, they were placed between amino acid residues 3 to 87, which were largely extended in the proteins. In large ACBPs, these ACBD were located near the C-terminal of the proteins, they were placed between residues 236 to 335. The ACBD in ankyrin repeats and kelch motif *Bn*ACBPs were localized near the N-terminal of the proteins. Ankyrin repeats ACBD were located between residues 90 to 182 in *Bn*ACBP1 (BnaA02g10270D and BnaC02g44810D) and between 101 to 190 in *Bn*ACBP2 (BnaA01g16660D and BnaC01g20440D). In kelch motif *Bn*ACBPs, they were located between residues 14 to 104 or 21 to 105 in *Bn*ACBP4 (Ais76194, Ais76195, Ais76196, Ais76199, Ais76200 and Ais76201) and between residues 26 to 104 in *Bn*ACBP5 (Ais76197 and Ais76198). N-terminal transmembrane domains could be found in ankyrin repeats and large *Bn*ACBPs as indicated in Fig 1. These structures were extended from amino acids 10 to 32 in *Bn*ACBP1 and 7 to 29 in *Bn*ACBP2 of ankyrin repeats *Bn*ACBPs. In large *Bn*ACBPs, the N-terminal transmembrane domains were found in residues 7 to 29 in two *Bn*ACBPs (BnaA01g13710D and BnaC01g16110D) but no transmembrane domain was detected in the two other *Bn*ACBPs (BnaA03g46540D and BnaC07g38820D). The additional conserved domains in ankyrin repeats and kelch motif *Bn*ACBPs made them different from the other classes. In fact, two ankyrin repeats domains were detected and they were extended between residues 251 to 283 and 284 to 316 in *Bn*ACBP1, and between residues 275 to 304 and 308 to 337 in *Bn*ACBP2. They belonged to PF00023. Furthermore, the kelch motif domains were placed in different positions. Three kelch motif domains were detected in three *Bn*ACBP4 (Ais76194, Ais76199, Ais76201) and in *Bn*ACBP5 (Ais76197 and Ais76198). Kelch domains were placed between residues 179 to 219, 292 to 345 and 344 to 394. They respectively belonged to PF13854, PF13964 and PF13418. However, three other *Bn*ACBP4 (Ais76196, Ais76195, Ais76200) contained only two kelch motif domains. The first domains were located between residues 183 to 239 and they belonged to PF13418, the second domains were located between residues 305 to 355 but their affiliation on the Pfam database was not found. These results indicated the difference of location and family of domains conserved in ACBPs, especially in kelch motif *Bn*ACBPs.

### Conserved residues of acyl-coA binding domains (ACBD)

**Fig 2:**
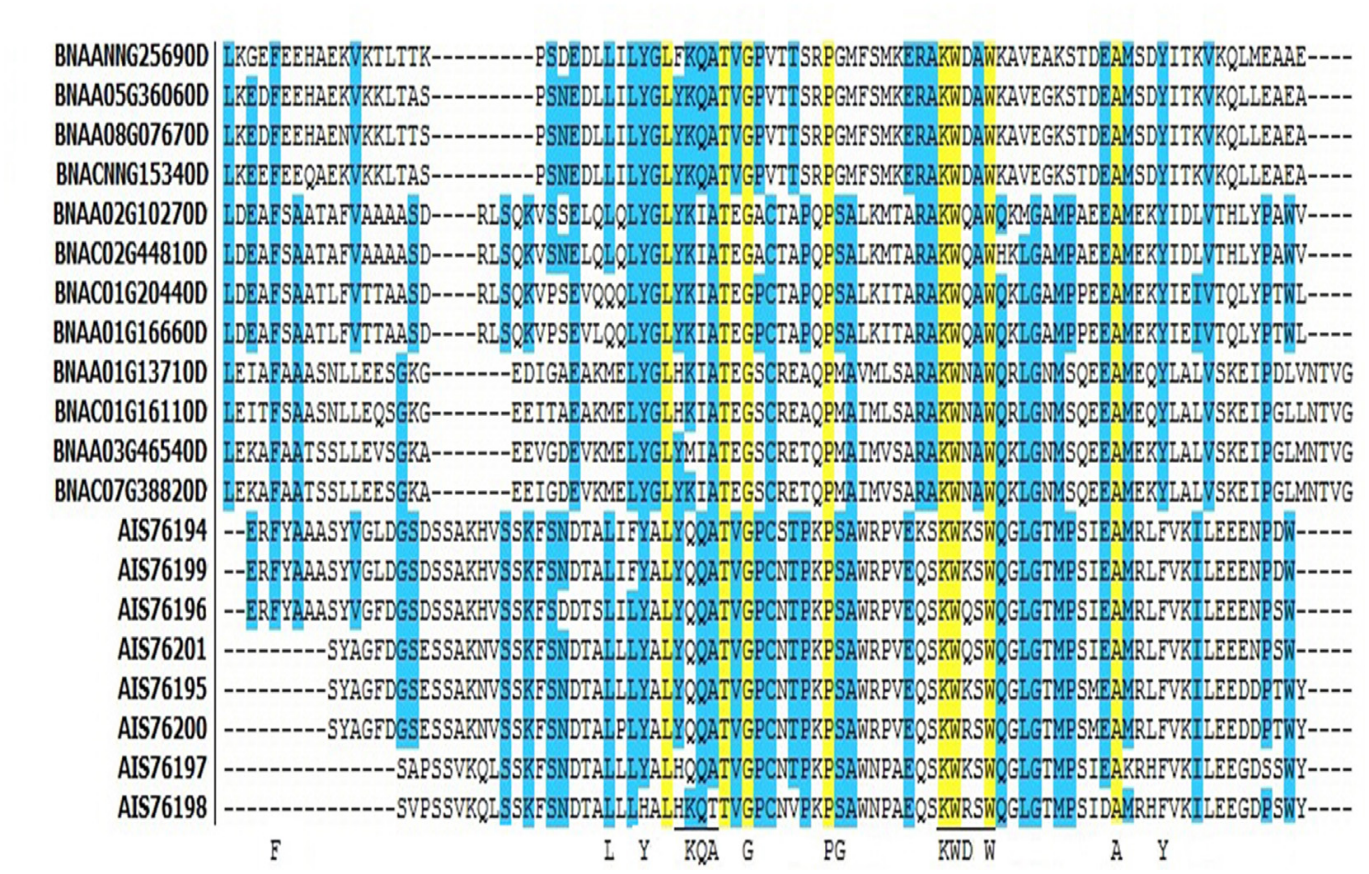
Acyl-coA binding domain alignment in *Bn*ACBPs. Alignment was generated using Vector NTI. Identical residues in all ACBPs are highlighted in yellow, identical residues in most of ACBPs are in blue. YKQA and KWDAW motifs correspond to the acyl-coA-binding site. The coenzyme-A head group-binding site are underlined. Capital letters indicate residues conserved in all ACBP of all species.

Conserved amino acid residues of ACBD in all four classes of *Bn*ACBPs were analyzed through alignment of all ACBPs (Fig 2). Identical residues in all four classes of *Bn*ACBPs corresponded to residues Leu-32, Thr-37, Gly-39, Pro-46, Lys-56, Trp-57, Trp-60, Ala-71 in small *Bn*ACBPs, Leu-135, Thr-140, Gly-142, Pro-149, Lys-159, Trp-160, Trp-163, Ala-174 in ankyrin repeats *Bn*ACBPs, Leu-267, Thr-272, Gly-274, Pro-281, Lys-291, Trp-292, Trp-295, Ala-306 in large *Bn*ACBPs and Leu-50, Thr-55, Gly-57, Pro-64, Lys-74, Trp-75, Trp-78, Ala-89 in kelch motif *Bn*ACBPs. In addition, some residues were conserved in almost all of the *Bn*ACBPs. These findings suggested eight amino acid residues conserved in all ACBD of *Bn*ACBPs.

### Predicted secondary structure of *Bn*ACBPs

**Fig 3:**
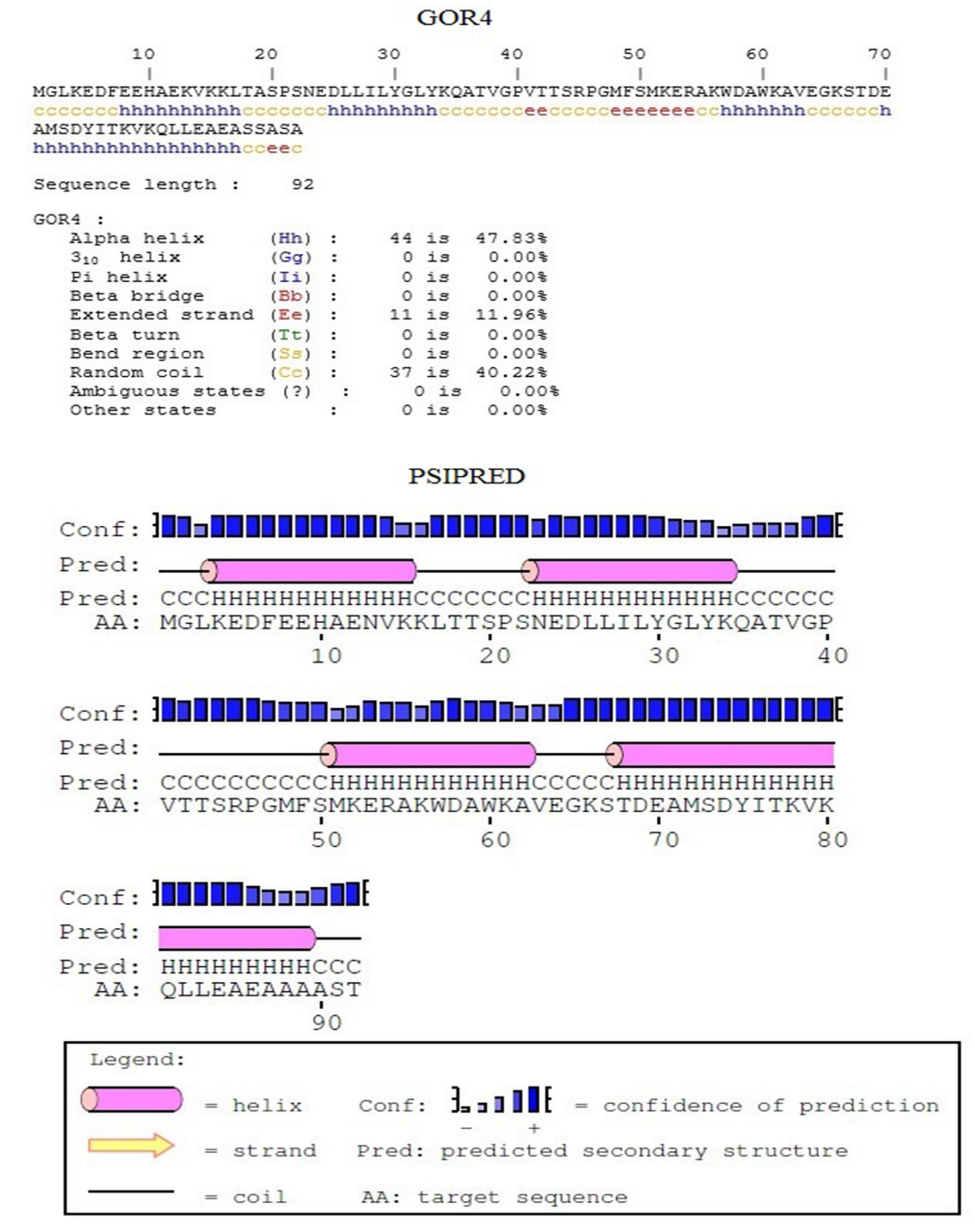
Predicted secondary structure of *Bn*ACBPs. Structures were predicted using GOR4 and PSIPRED. Pictures were reproduced from the server. Predicted structure of Small *Bn*ACBP is presented. Those of ankyrin repeats, large and kelch motif *Bn*ACBPs are in S1, S2 and S3 Figs, respectively.

To predict the secondary structure of *Bn*ACBPs, analysis was carried out using GOR4 and PSIPRED. Predictions of structure from GOR4 and PSIPRED were relatively the same. Predicted structures representing those of small ACBPs are shown in Fig 3. In small *Bn*ACBPs, they contained four helixes spaced out by coils and some extended strands, ~47% were represented by alpha-helixes and ~40% were coils. Yet, ~11% of the protein had extended strand structure. In ankyrin repeats *Bn*ACBPs, alpha-helixes represented ~54% of the protein (S1 Fig), coils and extended strands were ~36% and ~9%, respectively. Large *Bn*ACBPs' predicted secondary structure is shown in S2 Fig, they were mainly composed of ~49% of alpha-helixes, with ~39% of coils and ~10% of extended strands. However, coils were the main part of the kelch motifs *Bn*ACBPs with a percentage of ~48%. Strands formed ~21% of the proteins (S3 Fig). These results indicated that alpha-helixes composed a significant part of these *Bn*ACBPs structures.

### Predicted three dimensional structure of *Bn*ACBPs

**Fig 4:**
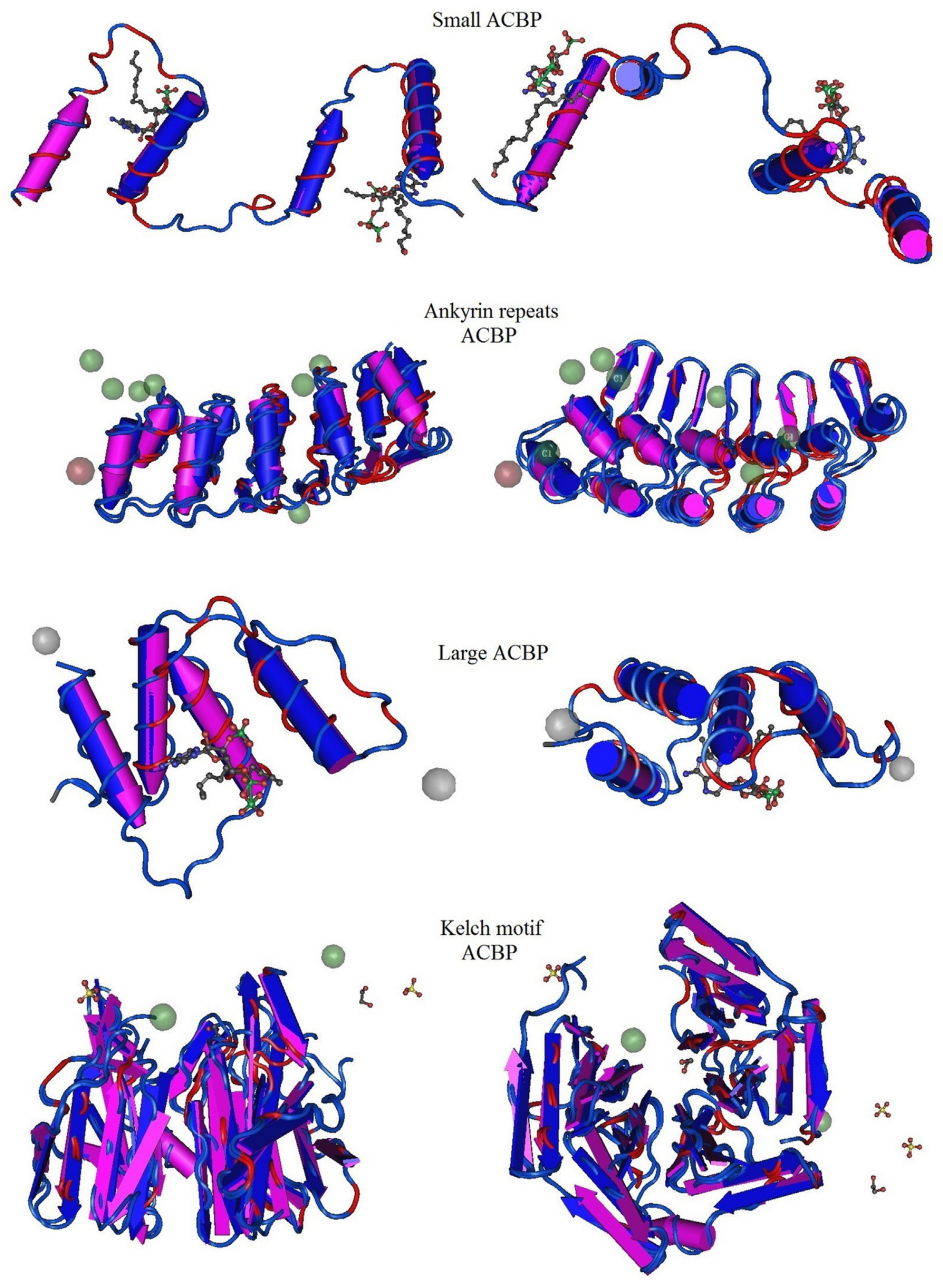
3D alignment between *Bn*ACBPs and their structure neighbors. The models were generated from Phyre2 with 100% of confidence. A part of each ACBP could be modeled: small ACBP (95%), ankyrin repeats ACBP (51%), large ACBP (23%), kelch motif ACBP(59%). Structure neighbors and 3D alignments were generated from VAST. Entire chains including only the aligned residues are shown. Red labels indicate amino acid residues that are identical in *Bn*ACBPs and their neighbors.

**Fig 5:**
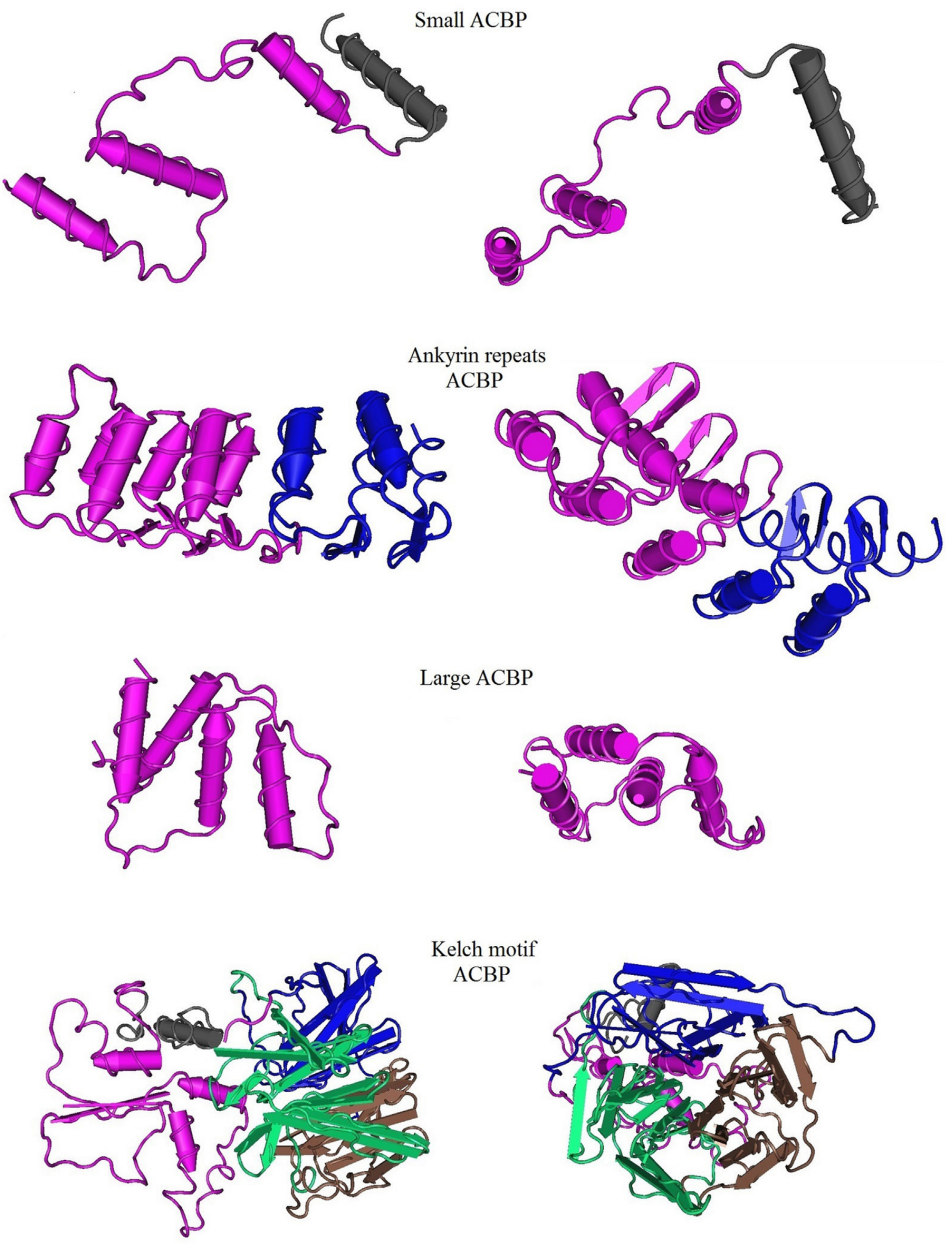
Predictive 3D domain structure of *Bn*ACBPs. The model were generated from Phyre2 with 100% of confidence. Conserved domain analysis were highlighted using VAST. Front view and top view are presented for each class of ACBP. Small ACBP: 95% of the protein are with presented ACBD highlighted in pink. Ankyrin repeats ACBP: 51% of the protein are shown with ACBD in pink and the ankyrin domain in blue. Large ACBP: 23% of the protein are shown, with ACBD in pink. Kelch motif ACBP: 59% of the protein are shown, ACBD are highlighted in pink, kelch domains are in blue, brown and green.

Three-dimensional structures of *Bn*ACBPs were predicted and modeled from Phyre2 database. The top models with 100% of confidence were considered. The acquired models which represented a part of the total proteins were then submitted to VAST analysis, on one hand, to compare with structure neighbors from the Protein Data Bank in medium redundancy and, on other hand, to highlight the conserved domains of each protein using a Cn3D macromolecular structure viewer. The highlighted domains were analyzed separately in SMART and annotated using Pfam. The three-dimensional profiles exposing the alignment between *Bn*ACBPs and the neighbors are shown in Fig 4. Sequence alignments are shown on S4 Fig. Similarly, the 3D profiles highlighting the conserved domains in each *Bn*ACBP are shown in Fig 5. The small *Bn*ACBP profile was built from the ACBD of Human ACBP7 associated with palmitoyl-coA (ID: 3EPY). It shared 48% of identity with small *Bn*ACBP and 95% of the protein was covered in this profile. In this 3D alignment, 87 residues were aligned with those of their neighbors. Otherwise, the 3D domain structure of the small *Bn*ACBP illustrated four alpha-helixes, in which three were labeled to contain one domain. This domain was located between residues 1 to 66, analysis with SMART indicated that it belonged to ACBD, annotated PF00887. The 3D structure of ankyrin repeats *Bn*ACBP was modeled from a crystal structure of ankb 24 ankyrin repeats, in complex with ankr2 (ID: 4RLV) with 25% of identity, and 51% of the ankyrin repeats *Bn*ACBP were covered. However, VAST alignment analysis presents the D34 Region of Human Ankyrin-r and Linker (ID: 1N11) as the structure neighbor. The 3D structure had 173 residues aligned with those of its neighbor. The profile of the 3D structure showed eight alpha-helixes with two different domains: the first domain implied six alpha-helixes were extended in residues 1 to 101, SMART analysis revealed that residues 1 to 57 of this first domain belonged to ACBD. The second domain implied two alpha-helixes with extended strands located on residues 102 to 176, with SMART analysis revealing two ankyrin domains belonging to PF00023. Since a part of the protein could be modeled, this part might correspond to those holding the ACBD and the ankyrin domains. Otherwise, transmembrane helixes were predicted on the ankyrin repeats *Bn*ACBP. In the large *Bn*ACBP, 3D structure displayed only 23% of the protein modeled with an ACBP from *Plasmodium falciparum* (ID: 1HBK), which shared the 25% of identity with them. Yet, 58% of the protein sequence were disordered. Currently, 88 residues were aligned with the neighboring residues. As in small *Bn*ACBP, four alpha-helixes form the 3-D structure of the large *Bn*ACBP that could be modeled. These helixes were in residues 1 to 88 and the related domain belonged to ACBD. Finally, the 3D structure of the kelch motif *Bn*ACBP was modeled from the trna wybutosine synthesizing enzyme2 TYW4 (ID: 2ZWA) which shared 17% of their identity. The confidence level was still 100% however the model covered only 59% of the total protein. As in ankyrin repeats *Bn*ACBPs, the VAST alignment analysis exposed another protein for the structure neighbor. The kelch motif *Bn*ACBP was then aligned with a crystal structure of the human Klhl3 kelch domain in complex with a Wnk4 peptide (ID: 4CH9), with 232 residues aligned with those of its neighbor. The profile of the 3D domain structure showed four alpha-helixes in which three were in residues 31 to 131, corresponding to the ACBD in SMART analysis. Besides, three other domains were presented in a sheet structure. They were located on residues 132 to 199, 200 to 304 and 305 to 431, respectively. SMART analysis showed that they contained kelch motif domains, with PF07646, PF13418 and PF13854, respectively. These results still confirmed our previous findings in which alpha-helixes composed the main part of the proteins.

### *Bn*ACBPs' Ramachandran plots

**Fig 6:**
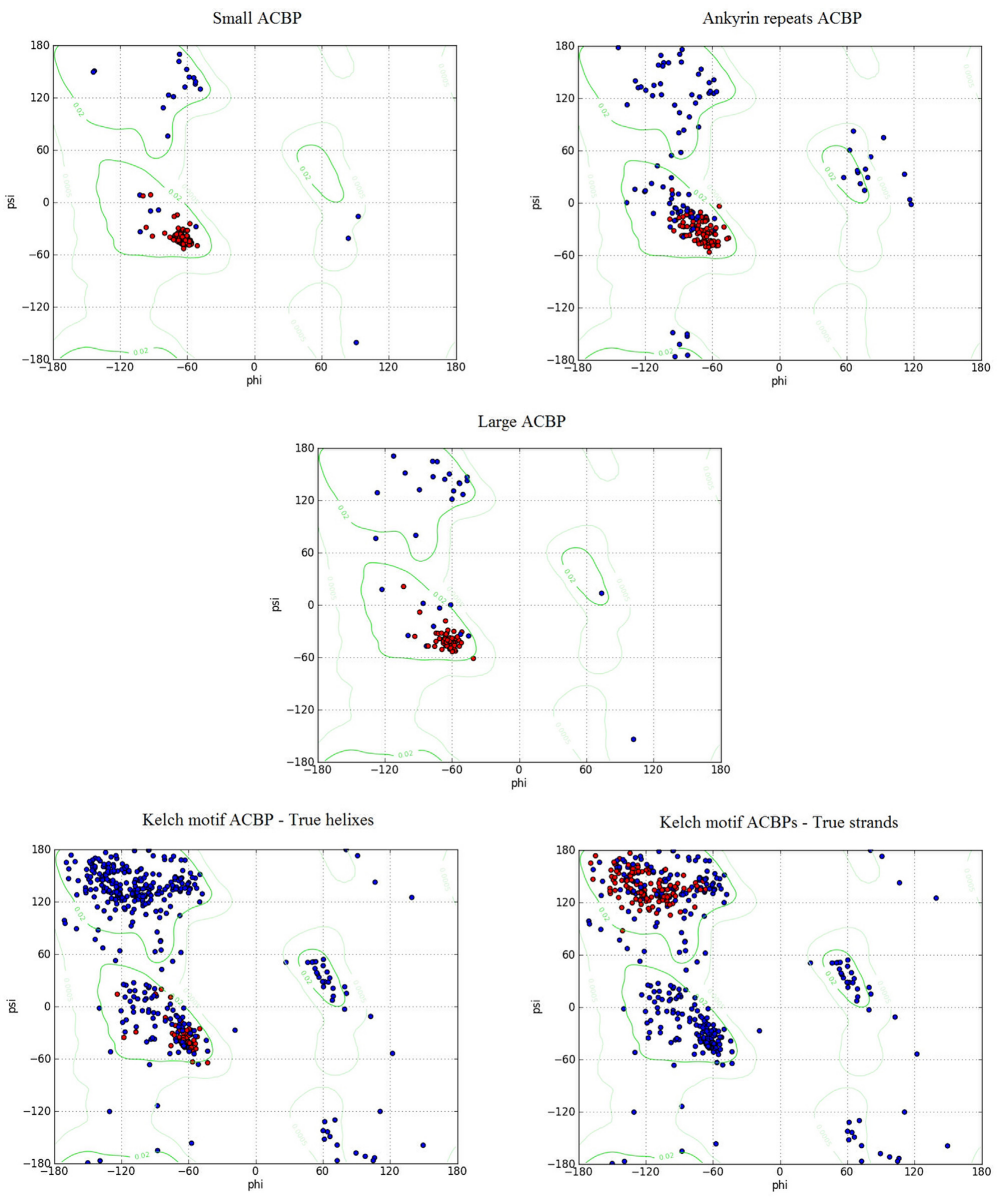
Ramachandran plot of *Bn*ACBPs. The plots were generated using UCSF Chimera, concerning all amino acids except for Glycine and Proline. Red plots indicate true helixes attribute (in all ACBPs) or true strands attribute (in kelch motif ACBP only), according to the region they are placed.

Ramachandran plots were generated from UCSF Chimera for each class of *Bn*ACBPs, in order to validate previous structure predictions. The plots are presented in Fig 6. The analyses were made from the modeled *Bn*ACBP proteins obtained from Phyre2 and the Ramachandran plots concerned all amino acids except Pro and Gly. In fact, Proline has a cyclic side chain that could restrict phi values to angles around -60. Glycine has no side chain so it covers a large range of area in the plot. Results might be indistinguishable if Gly and Pro were also considered for the analysis (detailed explanation is provided by Lovell et al., 2003 [35]). The attributes of residues for true helixes and true strands were chosen to assign probabilities. True helix attributes analysis implied 62/87 residues (71.26%), 77/173 residues (44.50%), 57/88 residues (64.77%) and 53/391 residues (13.55%) respectively in small, ankyrin repeats, large and kelch motif *Bn*ACBPs. However, true strand attributes involved only the kelch motif *Bn*ACBP with 122/391 residues representing 31.20% of them. These findings indicated that alpha-helixes structure was dominant in these *Bn*ACBPs, except for the kelch motif *Bn*ACBPs in which the beta-strands structure was more significant.

### Subcellular localization prediction of *Bn*ACBPs

Subcellular localization of each *Bn*ACBP was predicted using two different tools, results are shown in Table 2. Analysis with TargetP 1.1 (S2 Table) showed that ankyrin repeats and large *Bn*ACBPs were secretory proteins. Small and kelch motif *Bn*ACBPs were predicted to be located in other undefined locations. MultiLoc2 showed that ankyrin repeats and large *Bn*ACBPs were mainly located in endoplasmic reticulum, while small and kelch *Bn*ACBPs were mainly located in the cytoplasm (S3 Table). These results suggested that small and kelch motif ACBPs were located in the cytoplasm and the ankyrin repeats and large ACBPs were secretory proteins that were located in endoplasmic reticulum.

**Table 2:** Predictive subcellular localization of *Bn*ACBPs.

TYPE		TargetP 1.1	MultiLoc2
Small	BnaAnng25690D	Other	Cytoplasm
	BnaA05g36060D	Other	Cytoplasm
	BnaA08g07670D	Other	Cytoplasm
	BnaCnng15340D	Other	Cytoplasm
Ankyrin repeats	BnaA02g10270D	Secretory pathway	Endoplasmic reticulum
	BnaA01g16660D	Secretory pathway	Endoplasmic reticulum
	BnaC02g44810D	Secretory pathway	Endoplasmic reticulum
	BnaC01g20440D	Secretory pathway	Endoplasmic reticulum
Large	BnaA01g13710D	Secretory pathway	Endoplasmic reticulum
	BnaA03g46540D	Secretory pathway	Endoplasmic reticulum
	BnaC01g16110D	Secretory pathway	Endoplasmic reticulum
	BnaC07g38820D	Secretory pathway	Endoplasmic reticulum
Kelch motif	Ais76194	Other	Cytoplasm
	Ais76195	Other	Cytoplasm
	Ais76196	Other	Cytoplasm
	Ais76197	Other	Cytoplasm
	Ais76198	Other	Cytoplasm
	Ais76199	Other	Cytoplasm
	Ais76200	Other	Cytoplasm
	Ais76201	Other	Cytoplasm

Details of analyses are in S2 and S3 Tables.

## Discussion

### Physicochemical parameters of *Bn*ACBPs are similar to *At*ACBPs

Identification of *Brassica* ACBPs was based on homology to *At*ACBPs. The weights of ACBPs were approximately 10 KDa in small *Bn*ACBPs, 38 KDa in ankyrin repeats *Bn*ACBPs, 39 KDa in large *Bn*ACBPs and 73 KDa in kelch motifs *Bn*ACBP. These weights were relatively similar to those of *A*. *thaliana* with 10.4 KDa for small *At*ACBPs, 37.5 KDa and 38.5 KDa for ankyrin repeats *At*ACBPs, 39.3 KDa for large *At*ACBPs, 73.2 KDa and 71 KDa for kelch motif *At*ACBPs [6, 7, 8, 9, 10, 11]. Moreover, earlier study revealed that the homologue 10 KDa ACBP in *B*. *napus* contained 92 amino acids [12], which is consistent with our findings. Intron-exon structures were conserved in small *Bn*ACBPs and in kelch motif *Bn*ACBPs, respectively with 3 introns and 4 exons in small *Bn*ACBPs and 17 introns and 18 exons in kelch motif *Bn*ACBPs as in *At*ACBPs [9]. Few insertions and deletions might occur within introns during the evolutionary period, resulting in uniformity within structure. However, in ankyrin repeats ACBPs that contained 5 introns and 6 exons in *At*ACBPs [9], a gain in introns was observed in BnaC01g20440D. The other three copies conserved the same intron-exon structure of ankyrin repeats *At*ACBPs. In large *Bn*ACBPs, one copy (BnaA01g13710D) conserved the intron-exon structure of the large *At*ACBP, with 3 introns and 4 exons [9]. The other three genes might have lost an intron during the evolutionary period as they now contain 2 introns and 3 exons. The pI, the hydropathicity and the charge of *Bn*ACBPs might be related to their amino acid composition. *Bn*ACBPs' pI was relatively acid (4.12 in large ACBPs to 5.4 in small ACBPs), similar to *At*ACBPs, which was estimated to 5.04 [6]. Given that hydropathicity values were inferior to 1, *Bn*ACBPs are proposed to be hydrophilic proteins [13]. The amino acid composition of each *Bn*ACBPs showed that these proteins had more negative residues than positive residues. *Bn*ACBPs are then hydrophilic and negatively charged proteins, along with their acidic pI characteristics. The pI of proteins are related to their length and subcellular localization [14]. In our analysis, it is unclear how to define the correlation between length and pI as *Bn*ACBPs have all acidic pI even if their lengths are very different. These predictions were made using the ExPaSy—ProtParam, tool which allowed a computational analysis related to various physicochemical parameters for a given protein including the molecular weight, theoretical pI, amino acid composition, grand average of hydropathicity and many more [22]. Otherwise, close similarity in physicochemical parameters is not surprising in the two *Brassicaceae* genera.

### Difference within residues and domains emerged from evolution, leading into the difference in binding affinities and significant functions

The ACBD is the core of ACBPs that label them into their acknowledged functions of acyl-coA transporters with high affinity [9, 10, 16]. ACBPs progressed from the same ancestor but from duplication and rearrangement events emerge their difference within structure and their divergence into separate classes [1, 17, 18]. As presented in our study, the size and the amino acid composition were not uniform in *Bn*ACBPs, especially within their ACBD. Moreover, additional domain structure could be found as in ankyrin repeats and kelch motif ACBPs. The ACBD was compared in all *Bn*ACBPs. Eight amino acid residues corresponding to Leu-32, Thr-37, Gly-39, Pro-46, Lys-56, Trp-57, Trp-60 and Ala-71 in small *Bn*ACBP were conserved in all of them and a set of about forty amino acid residues were conserved in most of them. Comparison of 10 KDa ACBPs in all species including the 10 KDa *Bn*ACBP indicated fifteen highly conserved residues, corresponding to Phe-7, Leu-27, Tyr-30, Lys-34, Glu-35, Ala-36, Gly-39, Pro-46, Gly-47, Lys-56, Trp-57, Asp-58, Trp-60, Ala-71 and Tyr-75 similarly in *A*. *thaliana* and *B*. *napus* [6]. In our findings, small *Bn*ACBPs only conserved these fifteen residues; the other classes lacked one or more of these residues. Mutation that occurred during evolution might result in alteration of these conserved residues. These binding sites were those presented previously as divided into three subsites: one for the acyl part of the ligand, one for the adenine ring and one for the 3’-phosphate [14]. Otherwise, ACBD within the four classes of ACBPs were compared in *A*. *thaliana*: YKQA and KWDAW motifs related to acyl-coA-binding site and the coenzyme A head group-binding site were proposed to be conserved in all four classes [12, 13]. Currently in *Bn*ACBPs, they showed some differences. *B*. *napus* ACBD residues could vary which possibly enhanced them with dissimilar binding affinities with those of *At*ACBPs. In all ACBPs, the ACBD belonged to the same domain family (PF00887), but a difference was then perceived in amino acid residues, which might explain the difference in their binding affinity. Moreover, changes in amino acids and/or their positions could affect the binding ability of *A*. *thaliana* rACBPs [10, 15]. In our findings, ACBD in small ACBPs were located in residues 3 to 87, however they was demonstrated to be located in positions 3 to 82 in *A*. *thaliana* [6]. Small *At*ACBPs could bind linoleoyl-coA [11] whereas small *Bn*ACBPs could bind oleoyl-coA and palmitoyl-coA in 1/1 ratio [36]. Besides, recombinant small *Bn*ACBPs could reverse free oleoyl-coA inhibition of glucose-6-phosphate uptake from roots into plastids [37]. Small *At*ACBPs could be involved in plant freezing tolerance [38, 39, 40]. Large *At*ACBPs' binding domain was located on residues 231 to 314 [10], but our results indicated the position between 236 to 335 in *Bn*ACBPs. Large *At*ACBPs could bind arachidonyl-coA [10] and they could be involved in multiple biological functions as in plant defense signaling during fungal infection [41], circadian regulation [42], and response to hypoxia [43]. Transmembrane domain could be detected on large *At*ACBPs, which could overlap with signal peptides [10]. Strangely, the analysis from SMART database indicated the lack of these transmembrane domains in two large *Bn*ACBPs, structure that should target proteins into endomembrane and extracellular space [10, 44]. Similarly, in ankyrin repeats *At*ACBPs transmembrane domains were detected; deletion of these transmembrane domains could not target the proteins into the plasma membrane [45, 46]. In these ankyrin repeats *Bn*ACBPs, the ACBD were located on residue 90 to 182 in *Bn*ACBP1 and 101 to 190 in *Bn*ACBP2. They were defined to be placed respectively on positions 94 to 180 and 104 to 190 in *At*ACBPs [8, 45]. Ankyrin repeats *At*ACBPs could bind linoleoyl-CoA and linolenoyl-CoA esters [47]. Besides, the C-terminal ankyrins repeats of these ACBPs could enhance them to additional functions. Basically, ankyrins repeats proteins are among the most widespread structural motifs that could mediate protein-protein interactions [48]. They commonly have 33 amino acid residues sequence motif ensuring diverse functions: transcription initiation, cytoskeletal integrity, ion transport, cell signaling and cell cycle regulation [48, 49]. They are responsible for targeting structurally diverse proteins to specialized compartments as the endoplasmic reticulum or the plasma membrane [50]. Four to six repeats are commonly found in these structures, although thirty-four repeats could be found in other species [51]. In *A*. *thaliana*, ankyrin repeats ACBPs could be involved in many important biological functions as *A*. *thaliana* ethylene-responsive element-binding protein (*At*EBP) or farnesylated protein 6 (*At*FP6) interactions [47, 52], heavy metal accumulation response [47], abscisic acid (ABA) signaling treatment [53, 54], steam cuticle formation [55], Pb (II) accumulation in roots [56], and drought tolerance [57]. In the current study, the ACBD in kelch motif *Bn*ACBPs were located on positions 14 to 104 or 21 to 105 in *Bn*ACBP4 and 26 to 104 in *Bn*ACBP5. In our previous study, using NCBI Batch CD-search, these ACBD were predicted to be located at 14 to 104 in *Bn*ACBP4 and 12 to 103 in *Bn*ACBP5. However, they were identified as located on positions 12 to 103 in *At*ACBP4 and 22 to 104 in *At*ACBP5 [9]. Incoherence between the results obtained from NCBI Batch CD-search analysis and SMART analysis is apparent. Kelch motif *At*ACBPs could bind oleoyl-coA with high affinity so they could suppress glucose-6-phosphate inhibition in presence of free oleoyl-coA [9]. Similarly to the ankyrin repeats ACBPs, these kelch motif ACBPs have these kelch motifs additional structure that enhances them with other biological functions, especially those resulted from protein-protein interaction [58]. In our study, two or three kelch domains were found in *Bn*ACBPs, they were located between residues 181 to 239, 292 to 356 and 344 to 394. However in our previous study, four or five kelch domains were detected, they were located between residues 181 to 219, 242 to 283, 303 to 354, 357 to 405 and 392 to 439. Again, dissimilarity in results obtained from SMART and NCBI conserved domain analysis is evident. Ais76195, Ais76196 and Ais76201 kelch motif families were dissimilar to those of the other proteins, which was not expected, considering the alignment of kelch motifs *Bn*ACBPs (not shown) that emphasized very high similarity in amino acid sequences. Previous analysis using Batch-CD search from NCBI, however showed that Ais76195, Ais76196 and Ais76201 kelch motifs were very similar to the other proteins. Batch-CD search might be more reliable than SMART as tool for conserved domain analysis. In fact, kelch motifs contain about 44 to 55 amino acids, they are ancient and widely dispersed during evolution, so they could be found in proteins of eukaryotes and bacteria; they could be found in four to seven copies in *Drosophila* proteins [59, 60, 61]. Kelch-repeat proteins might be associated with actin cytoskeleton or might affect the organization of cytoskeletal, plasma membrane or organelle structures [61]. They were also recognized in galactose oxidase of *Dactylium dendroide* [62]. In *At*ACBPs, both ankyrin repeats and kelch motif ACBPs interact with other proteins in response to biotic and abiotic stress factors [47, 52, 63, 64, 65]. For instance, kelch motif *At*ACBP4 were involved in *At*EBP-mediated defense, similarly to ankyrin repeats *At*ACBP2, probably through ethylene or jasmonate signaling [64], but also in Pb (II) accumulation in roots [56]. All these functions might be found in *Bn*ACBPs, as structures are closely similar to *At*ACBPs. It might be possible that the functions are similar as structures are also similar, but loss or gain of new functions should be expected. Overall, difference in functions of each class of ACBPs might be the reflection of their difference within structure.

### Alpha-helixes compose the major structure of *Bn*ACBPs

The predictions on secondary structure of *Bn*ACBPs were made using GOR4 and PSIPRED. In both analysis, alpha-helixes were predicted to be the main part of the protein structure in small, ankyrin repeats and large *Bn*ACBPs (~47%, ~54% and ~49%, respectively). Although the alpha-helixes were not the dominant structure in kelch motif *Bn*ACBPs (~30%), they were significantly displayed in the protein, but in the kelch motif domains. These alpha-helixes structures were more clearly displayed in the three dimensional structure predictions. In fact, the three dimensional structure of *Bn*ACBPs was predicted with Phyre2 to find the most confident template in which a model could be attributed to each class of *Bn*ACBPs. Confidence levels were 100%, indicating the relationship between the proposed templates with our proteins as a true homology. The overall folds shown were well adopted and the core of each protein could be modeled at high accuracy [28]. However, the identity shared with the respective model was very weak (17% in kelch motif ACBPs to 48% in small ACBPs) but with a very high confidence level, the proposed model could be still very useful [28]. Moreover, VAST allowed to highlight the conserved domains in each 3D structure of ACBPs, in use of Cn3D macromolecular structure viewer, but the conserved domain predicted was not consistent with those predicted in SMART. Moreover, the model covered a portion of ACBP so that the location of conserved domains was imprecise. VAST alignment analysis allowed finding their protein neighbor, resulting in alignment exposed in Fig 4. The neighbors are homologue proteins identified by a significant similarity score using the VAST algorithm, and based on a direct comparison of 3D structure [29, 66, 67]. Thus, distant and undetectable evolutionary relationships can be revealed giving possibility to emerge unsuspected functional properties [29]. In our analysis, structure neighbors suggested by Phyre2 and VAST were not similar for ankyrin repeats and kelch motifs *Bn*ACBPs. The additional protein-protein interactions site within these two classes of ACBP might lead to disagreement in structure neighbor suggestions, since the two other classes, which contain only the ACBD, were consistently suggested to the same structure neighbor. Otherwise, structure corresponding to the ACBD showed alpha-helixes shape in all *Bn*ACBPs. In fact, this structure was first elucidated in bovine ACBPs, in which four alpha-helixes held by hydrophobic interactions and showed an up-down-down-up direction were detected [13, 16]. The proposed structure in small *Bn*ACBPs covered 95% of the protein and the ACBD was largely extended. The part of the large *Bn*ACBPs that could be modeled (23% of the protein) represented the C-terminal ACBD, the protein might be an orphan with no sequence homologues [28]. In addition to the common ACBD that were conserved in the four classes of ACBPs, ankyrin repeats ACBPs and kelch motif ACBPs had these protein-protein interactions domains that made them different from the other classes of ACBPs. In fact, independent forms of these domains had their particular shape, but in ACBPs they were combined with the ACBD to generate these proposed models. Ankyrins in their ordinary shape are formed by 30% alpha-helixes [68, 69]. Ankyrin repeats have two alpha-helixes following a beta-hairpin loop in L-shape [70]. The structure is organized as following: multiple repeats create an inner core stabilized by helix-helix interactions, beta-hairpins create potential surfaces for interactions with targeted proteins, anti-parallel beta-sheets formed by hydrophobic bonds between repeats stabilize the protein. Thus, it was found that potential binding sites could be created by the exposed tips of beta-hairpins [69]. In kelch motifs, the conserved three-dimensional structure is composed of beta-propellers that contain tandem kelch motifs. A single blade of the propeller is formed by a four-stranded beta-sheet containing the kelch motif, and the series of blades lie twisted around a central axis [61]. Beta-propeller structure could be stabilized by interactions between both the N- and C-terminal regions of the domain [62], but one of them might be enough [61]. Coils and helixes in our secondary structure prediction represented the C-terminal kelch motif domains in *Bn*ACBPs. However, sheet structures were exposed from the three-dimensional structure prediction. These kelch motif domains were expected to have these beta-propeller structures. Inconsistency between secondary and tertiary structure predictions is apparent. As a structure validation, these *Bn*ACBPs were subjected to analysis of their polypeptide backbones, generating the Ramachandran plots. The Ramachandran plot confirmed that alpha-helix structure were foremost in these *Bn*ACBPs, which probably corresponded to the ACBD. As kelch proteins are generally in beta-propeller shape, Ramachandran plot could validate the significant extent of this structure in kelch motif *Bn*ACBPs. Experimental approaches are greatly needed to overcome uncertainty about the real shape of these *Bn*ACBPs. In any case, all of them have the helixes shape, and the beta-propeller added to the kelch motif *Bn*ACBPs is normal due to the beta-propeller original form of kelch proteins.

### Structure of *Bn*ACBP could define their subcellular localization

Subcellular localization of *Bn*ACBPs was predicted from tools of two websites each using a different algorithm. On one hand, TargetP 1.1 could predict the location of proteins in the basis of the predicted presence of N-terminal pre-sequences: chloroplast transit peptide (cTP), mitochondrial targeting peptide (mTP) or secretory pathway signal peptide (SP) [33]. On the other hand, MultiLoc2 could reveal more precise results, as it operates in a large scale providing more robustness and a higher accuracy within analysis [34]. Small and kelch motif *At*ACBPs were predicted to be located in the cytoplasm [6, 9], this is consistent with the location of *Bn*ACBPs predicted from MultiLoc2. Ankyrin repeats *At*ACBPs are membrane-associated proteins found in plasma membrane and endoplasmic reticulum [7, 9]. Similarly, analysis revealed by MultiLoc2 indicated the localization of ankyrin repeats *Bn*ACBPs predominantly in endoplasmic reticulum. As well, large *Bn*ACBPs were predicted to be involved in the secretory pathway and localized in endoplasmic reticulum. However, extracellular localization of large ACBPs was clearly demonstrated in *A*. *thaliana* [10]. Structure of a protein might be related to its subcellular localization: the presence of the N-terminal transmembrane domains in large ACBPs and ankyrin repeats ACBPs might explain their subcellular localization, given that transmembrane domain knock-out could fail *At*ACBP1/*At*ACBP2 to be targeted in plasma membrane or endoplasmic reticulum [44, 46]. Moreover, a study on *A*. *thaliana* ankyrin repeats containing proteins showed that they were mainly localized in the membrane of endoplasmic reticulum [71]. Similarly, kelch motif proteins were shown to have an intracellular or extracellular localization, or located in the cell surface [61]. Thus, structure and subcellular localization are related.

## Conclusions

This study aimed to predict the structure of ACBPs in *B*. *napus*. Indeed, investigation on ACBP structure was made since 1993 by Kragelund et al. [13], but was unfortunately limited to the small single domain ACBPs. Our study focused on all the classes of ACBPs (single and multi-domains proteins) in *B*. *napus*. It is confident that structure could define functions and the location where these functions are fulfilled. Nevertheless, function could not define structure, as many proteins of dissimilar structure might have the same function. There might be some amino acids keys common to all protein of the same functions, but this needs to be demonstrated. Close similarity in ACBP structure between *A*. *thaliana* and *B*. *napus* is expected, as they belong to the same family. Thus, similar functions could be expected but loss or gain of new ones might be probable. Moreover, results given by software or databases show some dissimilarity. Experimental approaches are then required to affirm real statements.

## Supporting Information

S1 Fig Predicted secondary structure of ankyrin repeats *Bn*ACBPs. Result shown is from GOR4 prediction analysis. Prediction from PSIPRED is relatively the same.
(TIF)

S2 Fig Predicted secondary structure of large *Bn*ACBPs. Result shown is from GOR4 prediction analysis. Prediction from PSIPRED is relatively the same.
(TIF)

S3 Fig Predicted secondary structure of kelch motif *Bn*ACBPs. Result shown is from GOR4 prediction analysis. Prediction from PSIPRED is relatively the same.
(TIF)

S4 Fig VAST alignment of *Bn*ACBPs. VS74 represent the *Bn*ACBPs.
(TIF)

S1 Table Amino acid composition of *Bn*ACBPs. (XLSX)

S2 Table Predicted subcellular localization of *Bn*ACBPs—TargetP 1.1 analysis. (XLSX)

S3 Table Predicted subcellular localization of *Bn*ACBPs—MultiLoc2. (XLSX)
