# Supplementary figures and images for: Computational Prediction of acyl-coA Binding Proteins Structure in Brassica napus
Source: PLoS One. 2015 Jun 11;10(6):e0129650. doi: 10.1371/journal.pone.0129650 (PMC4465970; doi:10.1371/journal.pone.0129650)

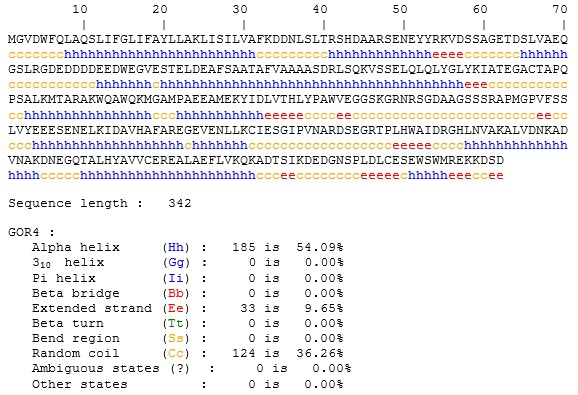

Supplement: S1 Fig — Result shown is from GOR4 prediction analysis. Prediction from PSIPRED is relatively the same. (TIF) [file pone.0129650.s001.tif]

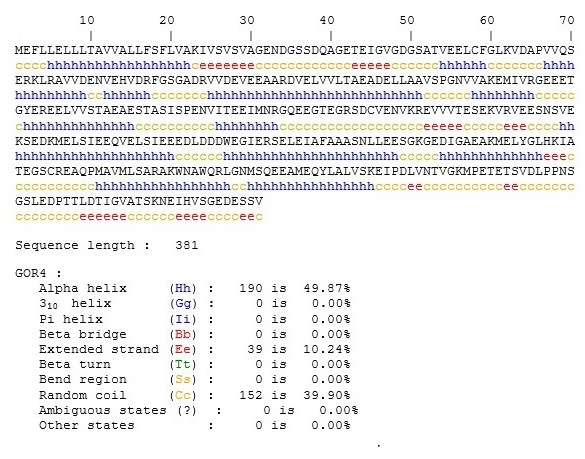

Supplement: S2 Fig — Result shown is from GOR4 prediction analysis. Prediction from PSIPRED is relatively the same. (TIF) [file pone.0129650.s002.tif]

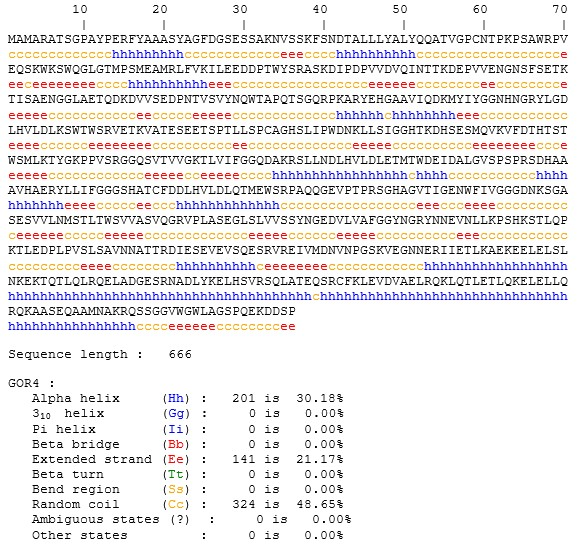

Supplement: S3 Fig — Result shown is from GOR4 prediction analysis. Prediction from PSIPRED is relatively the same. (TIF) [file pone.0129650.s003.tif]

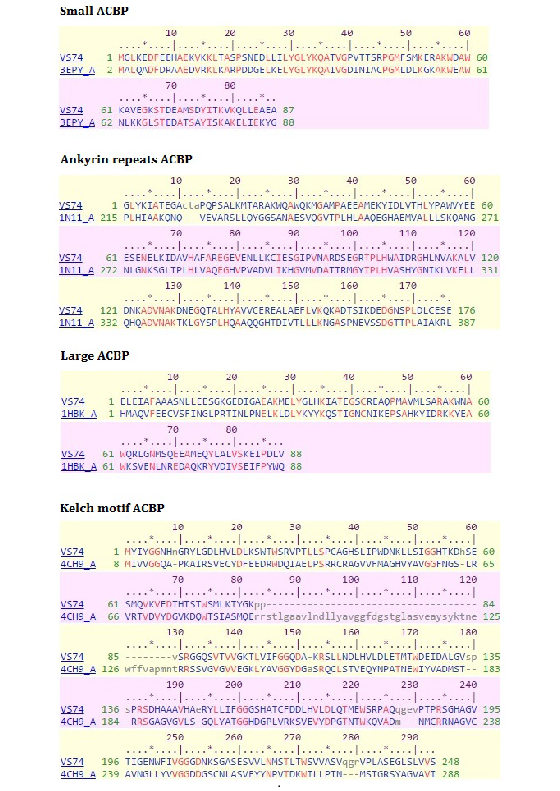

Supplement: S4 Fig — VS74 represent the BnACBPs. (TIF) [file pone.0129650.s004.tif]
